# Ridge Regression Method and Bayesian Estimators under Composite LINEX Loss Function to Estimate the Shape Parameter in Lomax Distribution

**DOI:** 10.1155/2022/1200611

**Published:** 2022-08-29

**Authors:** Mansour F. Yassen, Fuad S. Al-Duais, Mohammed M. A. Almazah

**Affiliations:** ^1^Mathematics Department, College of Humanities and Science in Al Aflaj, Prince Sattam Bin Abdulaziz University, Al-Kharj, Al Aflaj, Saudi Arabia; ^2^Department of Mathematics, College of Sciences and Arts (Muhyil), King Khalid University, Muhyil 61421, Saudi Arabia

## Abstract

In this paper, the Ridge Regression method is employed to estimate the shape parameter of the Lomax distribution (LD). In addition to that, the approaches of both classical and Bayesian are considered with several loss functions as a squared error (SELF), Linear Exponential (LLF), and Composite Linear Exponential (CLLF). As far as Bayesian estimators are concerned, informative and noninformative priors are used to estimate the shape parameter. To examine the performance of the Ridge Regression method, we compared it with classical estimators which included Maximum Likelihood, Ordinary Least Squares, Uniformly Minimum Variance Unbiased Estimator, and Median Method as well as Bayesian estimators. Monte Carlo simulation compares these estimators with respect to the Mean Square Error criteria (MSE's). The result of the simulation mentioned that the Ridge Regression method is promising and can be used in a real environment. where it revealed better performance the than Ordinary Least Squares method for estimating shape parameter.

## 1. Introduction

Ridge Regression is a popular parameter estimation method for analyzing multiple regression data which has multicollinearity. When Multicollinearity happens, Least squares estimates are unbiased, but their variances are large. As a result, the estimator of the Ordinary Least Squares Method (OLS) becomes far from the true value. Ridge Regression decreases the standard errors when a degree of bias is added to the Regression Estimates. Several authors have addressed Ridge Regression as in [1–10].

The Lomax distribution (LD) is a proposed distribution of the Pareto distribution of type II; it was used to obtain a good model for biomedical problems. Also, it is an important model for modeling failure times. The Lomax distribution was used as a stochastic model with a decreasing failure rate for the operating times of the electronic vehicles under study. It was also used in studies related to income and studies related to the size of cities. As well as being a useful model in studying queuing theory and in analyzing data related to biostatistics.

Many theoretical and statistician's studies have given great interest to estimating the parameter and survival analysis of LD.

Al-Noor and Alwan [11] compared the Nonbayesian, Bayesian, and Empirical Bayes estimate for the parameters of the LD by considering the symmetric and asymmetric loss functions. Al-Duais and Hmood [12] compared the Bayesian estimators and Classical estimators to estimate the parameter and survival analysis depending on record values and by considering the SELF, LLF, and WLLF. Ellah [13] estimated the parameter, reliability, and hazard function of the LD by applying the Bayesian estimators and Classical estimators based on record values by considering the SELF and LLF. Asl et al. [14] applied the Bayesian estimators and Classical estimators of prediction on unidentified parameters of an LD based on a progressively type-I hybrid censoring scheme. Mohie El-Din et al. [15] studied the Classical estimations and Bayesian estimations of the LD based on progressively type-II censored samples and by considering symmetric (SELF) and asymmetric (LLF and GELF), Okasha [16] estimated the parameters, and survival analysis of the LD by applying the E-Bayesian estimators and Bayesian estimators under type-II censored data and by regarding the balanced squared error loss function. Liu and Zhang [17] studied the Bayesian and E-Bayesian estimations of the LD Based on the Generalized Type-I Hybrid Censoring Scheme. to estimate the unknown parameter of LD and by considering SELF and LLF to estimate the parameter and reliability function. Al-Bossly [18] developed a compound LINEX loss function (CLLF) to estimate the shape parameter of the Lomax distribution utilizing the E-Bayes and Bayes estimation methods for the distributional parameters of the LD.

In the current study, Ridge regression was employed to estimate the shape parameter of LD and compare it with the classical estimators which included Maximum Likelihood, Ordinary Least Squares, Uniformly Minimum Variance Unbiased Estimator, and Median Method as well as Bayesian estimators. The uniqueness of this work comes from the fact that, to date, no attempt has been made to estimate the shape parameter of the LD using the method of Ridge regression.

The pdf of LD is given as follows [19]:

(1) $$\begin{array}{c} f\left(x;\vartheta ,\delta \right)=\left\{\begin{array}{cc} \frac{\vartheta }{\delta }{\left(1+\frac{x}{\delta }\right)}^{-\left(\vartheta +1\right)}; & x\geq 0;\vartheta ,\delta >0,\\ 0; & o.w, \end{array}\right. \end{array}$$

where *x* is a random variable, and *δ* > 0, *ϑ* > 0 are the scale and shape parameters, respectively.

The CDF and reliability function *R*(*t*) of (1) are given by the following equation:

(2) $$\begin{array}{c} F\left(x;\vartheta ,\delta \right)=1-{\left(1+\frac{x}{\delta }\right)}^{-\vartheta };\;x\geq 0;\;\vartheta ,\delta >0, \end{array}$$

(3) $$\begin{array}{c} R\left(t\right)={\left(1+\frac{t}{\delta }\right)}^{-\vartheta };\;t\geq 0;\vartheta ,\delta >0. \end{array}$$

## 2. Classical Methods of Estimation of Lomax Shape Parameter

The Classical methods selected for the comparative study are (i) Maximum Likelihood Estimator (MLE), (ii) Ordinary Least Squares Method (OLS), (iii) Ridge Regression method, (iv) Uniformly Minimum Variance Unbiased Estimator (UMVUE), and (v) Median Method (M.M).

### 2.1. MLE's of the Shape Parameter *ϑ*

Suppose that $\underline{x}=x_{1},x_{2},\ldots x_{n}$ is a random sample from the LD as in (1), then the $L\left(\underline{x}|\vartheta \right)$ for the sample observation will be as follows:

(4) $$\begin{array}{c} L\left(\underline{x}|\vartheta \right)=\prod_{i=1}^{n}\frac{\vartheta }{\delta }{\left(1+\frac{x}{\delta }\right)}^{-\left(\vartheta +1\right)}={\left(\frac{\vartheta }{\delta }\right)}^{n}\text{exp}\left[-T\left(\vartheta +1\right)\right], \end{array}$$

where *T*=∑_*i*=1_^*n*^In(1+*x*_*i*_/*δ*).

Log likelihood function

(5) $$\begin{array}{c} \ln\;L\left(\vartheta ,\delta \right)=n\ln\;\vartheta -n\ln\;\delta -\left(\vartheta +1\right)\sum_{i=1}^{n}\ln\left(1+\frac{x_{i}}{\delta }\right). \end{array}$$

The MLE's of *ϑ* denoted by ${\hat{\vartheta }}_{\text{MLE}}$ is given as follows:

(6) $$\begin{array}{c} {\hat{\vartheta }}_{MLE}=\frac{n}{\sum_{i=1}^{n}\text{ln}\left(1+x_{i}/\delta \right)}. \end{array}$$

### 2.2. Ordinary Least Squares Method (OLS)

The CDF in equation (2) satisfies

(7) $$\begin{array}{c} \text{ln}\left(1-F\left(x\right)\right)=-\vartheta \text{ln}\left(1+\frac{x}{\delta }\right)=-\vartheta \text{ln}\left(\delta +x\right)+\vartheta \text{ln}\delta . \end{array}$$

Now, suppose that *X*_1_, *X*_2_,……*X*_*n*_ form a random sample from LD defined by (1), and that *X*_(1)_ < *X*_(2)_ < …<*X*_(*n*)_ are the order statistics. With observed ordered observations *x*_(1)_ < *x*_(2)_ < …<*x*_(*n*)_ (2) gives the following equation:

(8) $$\begin{array}{c} \text{ln}\left(1-F\left(x_{\left(i\right)}\right)\right)=-\vartheta \text{ln}\left(1+\frac{x_{\left(i\right)}}{\delta }\right)=-\vartheta \text{ln}\left(\delta +x_{\left(i\right)}\right)+\vartheta \text{ln}\delta . \end{array}$$

(8) represents a simple linear regression function corresponding to *F*(*x*_(*i*)_)

(9) $$\begin{array}{c} Y_{i}=\alpha +\beta X_{i}+\varepsilon_{i}, \end{array}$$

where $Y_{i}=\text{ln}\left(1-{\hat{F}}_{i}\right)$ and ${\hat{F}}_{i}$*i* it is a point estimator of *F*(*x*_(*i*)_) many estimators for ${\hat{F}}_{i}$ are used.

For example, the Median Rank estimator ${\hat{F}}_{i}=\left(i-0.3\right)/\left(n+0.4\right)$ or ${\hat{F}}_{i}=\left(i-3/8\right)/\left(n+0.25\right)$, the mean rank estimator ${\hat{F}}_{i}=\;i/\left(n+1\right)$. where *i* denotes the *i*^*th*^ smallest value of *x*_(1)_, *x*_(2)_,…*x*_(*n*)_, *i*=1,2,…, *n*. *ε*_*i*_ is the random error with expected value *E*(*ε*_*i*_)=0. *X*_*i*_=ln(*δ*+*x*_(*i*)_), *β*=−*ϑ*, *α*=*ϑ*ln*δ*.

The estimates $\hat{\alpha }$ and $\hat{\beta }$ of the regression parameters, *α* and *β* minimize the function,

(10) $$\begin{array}{c} Q\left(\alpha ,\beta \right)={\sum_{i=1}^{n}\left(Y_{i}-\alpha -\beta \;\ln\left(\delta +x_{\left(i\right)}\right)\right)}^{2}. \end{array}$$

Therefore, the estimates ${\hat{\beta }}_{\text{OLS}}$ of the parameter, *β* is given by the following equation:

(11) $$\begin{array}{c} {\hat{\beta }}_{\text{OLS}}=\frac{n\sum_{i=1}^{n}\text{ln}\left(\delta +x_{\left(i\right)}\right)\text{ln}\left(1-{\hat{F}}_{i}\right)-\sum_{i=1}^{n}\ln\left(\delta +x_{\left(i\right)}\right)\sum_{i=1}^{n}\ln\left(1-{\hat{F}}_{i}\right)}{n\sum_{i=1}^{n}{\text{ln}}^{2}\left(\delta +x_{\left(i\right)}\right)-{\sum_{i=1}^{n}\text{ln}\left(\delta +x_{\left(i\right)}\right)}^{2}},\\ {\hat{\alpha }}_{\text{OLS}}=\;\frac{1}{n}\sum_{i=1}^{n}\ln\left(1-{\hat{F}}_{i}\right)-{\hat{\beta }}_{\text{OLS}}\frac{1}{n}\sum_{i=1}^{n}\ln\left(\delta +x_{\left(i\right)}\right). \end{array}$$

The estimate ${\hat{\vartheta }}_{\text{OLS}}$ of the parameter *ϑ* is given by the following equation:

(12) $$\begin{array}{c} {\hat{\vartheta }}_{\text{OLS}}=-{\hat{\beta }}_{\text{OLS}}. \end{array}$$

### 2.3. Ridge Regression Method

Ridge Regression estimates can be obtained by minimizing the function.

(13) $$\begin{array}{c} Q^{*}\left(\alpha ,\beta \right)=\sum_{i=1}^{n}\left(\ln\left(1-{\hat{F}}_{i}\right)-\alpha -\beta \;\ln\left(\delta +x_{\left(i\right)}\right)\right){\;}^{2}. \end{array}$$

According to the following constraint

(14) $$\begin{array}{c} \alpha^{2}+\beta^{2}=\phi , \end{array}$$

where *ϕ* is a definite positive constant.

The Lagrange multiples method requires that we derive the following:

(15) $$\begin{array}{c} L=\sum_{i=1}^{n}\left({\left(\ln\left(1-{\hat{F}}_{i}\right)-\alpha -\beta \;\ln\left(\delta +x_{\left(i\right)}\right)\;\right)}^{2}+\lambda \left(\alpha^{2}+\beta^{2}-\phi \right)\right),\\ \frac{\partial \;\ln\;L}{\partial \alpha }=-2\sum_{i=1}^{n}\left(\ln\left(1-{\hat{F}}_{i}\right)-\alpha -\beta \;\ln\left(\delta +x_{\left(i\right)}\right)\ln\left(\delta +x_{\left(i\right)}\right)\right)+2\alpha \lambda =0,\\ \frac{\partial \;\ln\;L}{\partial \beta }=-2\sum_{i=1}^{n}\left(\ln\left(1-{\hat{F}}_{i}\right)-\alpha -\beta \;\ln\left(\delta +x_{\left(i\right)}\right)\right)\ln\left(\delta +x_{\left(i\right)}\right)+2\beta \lambda . \end{array}$$

Therefore, the estimates ${\hat{\alpha }}_{\text{Rid}}$ and ${\hat{\beta }}_{\text{Rid}}$ of the parameters, *α* and *β* are given by the following equation:

(16) $$\begin{array}{c} {\hat{\beta }}_{\text{Rid}}=\frac{\left(n+\lambda \right)\sum_{i=1}^{n}\ln\left(\delta +x_{\left(i\right)}\right)\ln\left(1-{\hat{F}}_{i}\right)-\sum_{i=1}^{n}\ln\left(\delta +x_{\left(i\right)}\right)\sum_{i=1}^{n}\ln\left(1-{\hat{F}}_{i}\right)}{\left(n+\lambda \right)\sum_{i=1}^{n}\ln^{2}\left(\delta +x_{\left(i\right)}\right)\;-{\left(\sum_{i=1}^{n}\ln\left(\delta +x_{\left(i\right)}\right)\right)}^{2}},\\ {\hat{\alpha }}_{\text{Rid}}=\frac{\sum_{i=1}^{n}\ln\left(1-{\hat{F}}_{i}\right)-\beta \sum_{i=1}^{n}\ln\left(\delta +x_{\left(i\right)}\right)}{n+\lambda }, \end{array}$$

and

(17) $$\begin{array}{c} \lambda =\frac{\rho \sigma^{2}}{\beta^{\prime }\beta };0<\lambda <1, \end{array}$$

where *ρ* represents the number of parameter of the distribution and *β*′*β* represents the covariance matrix.

NOT when *λ*=0, we get the estimations of the OLS.

The Ridge Regression estimate of *ϑ* denoted by ${\hat{\vartheta }}_{\text{Rid}}$ is given as follows:

(18) $$\begin{array}{c} {\hat{\vartheta }}_{\text{Rid}}=-{\hat{\beta }}_{\text{Rid}}. \end{array}$$

### 2.4. UMVUE Estimator of the Shape Parameter *ϑ*

The pdf of LD belongs to the exponential family. Therefore, *T*=∑_*i*=1_^*n*^ln(1+(*x*_*i*_/*δ*)) is a complete sufficient statistic for *ϑ*. Then, depending on the theorem of Lehmann-Scheffe [20], the uniformly minimum variance unbiased estimator ${\hat{\vartheta }}_{\text{UMVUE}}$ of *ϑ*, may be given by the following equation:

(19) $$\begin{array}{c} {\hat{\vartheta }}_{\text{UMVUE}}=\frac{n-1}{\sum_{i=1}^{n}\ln\left(1+x_{i}/\delta \right)}. \end{array}$$

### 2.5. Median Method (M.M)

This method is dependent on the basis that the median divides the data into two equal parts

(20) $$\begin{array}{c} F\left(x_{\text{med}}\right)=0.5. \end{array}$$

By substituting into the cumulative distribution function defined by (2). The equation will become

(21) $$\begin{array}{c} 1-{\left(1+\frac{x_{\text{med}}}{\delta }\right)}^{-\vartheta }=0.5. \end{array}$$

Therefore, the estimates ${\hat{\vartheta }}_{\text{Med}}$ of *ϑ*, can be obtained as follows:

(22) $$\begin{array}{c} {\hat{\vartheta }}_{\text{Med}}=\frac{-\log\left(0.5\right)}{\text{log}\left(1+x_{\text{med}}/\delta \right)}, \end{array}$$

where *x*_med_ is the median of the data.

## 3. Prior and Posterior Density Functions

### 3.1. Prior Distribution

The Bays estimators demand an appropriate selection of priors for the parameter. If we do not have sufficient knowledge about the parameter, in this case, the noninformative priors are better chosen. Or else, it is desirable to use informative priors. In this research, we study both types of priors: informative priors and noninformative priors.

#### 3.1.1. Non-Informative Prior

Let us assume that *ϑ* has noninformative prior density defined as using extended Jeffrey's prior *h*_1_(*ϑ*) which is given by the following equation:

(23) $$\begin{array}{c} h_{1}\left(\vartheta \right)\propto {\left[I\left(\vartheta \right)\right]}^{c};\;c>0, \end{array}$$

where *I*(*ϑ*) represented Fisher information matrix which defined as follows:

(24) $$\begin{array}{c} I\left(\vartheta \right)=-nE\left[\frac{\partial^{2}\text{log}f\left(x;\vartheta ,\delta \right)}{\partial \vartheta }\right],\\ h_{1}\left(\vartheta \right)=\frac{1}{\vartheta^{2c}};c>0. \end{array}$$

#### 3.1.2. Informative Priors (The Natural Conjugate Prior)

In this work, three types of prior distributions were used to study the effect of the different prior distributions on a Bayesian estimate of *ϑ*.

(a) Chi-squared prior
  (25) $$\begin{array}{c} h_{2}\left(\vartheta \right)=\frac{d^{k/2}}{2^{k/2}\Gamma \left(k/2\right)}\vartheta^{k/2-1}\exp\left[-\frac{d\vartheta }{2}\right];\;\vartheta >0;k,d>0. \end{array}$$
(b) Inverted levy prior
  (26) $$\begin{array}{c} h_{3}\left(\vartheta \right)=\sqrt{\frac{k}{2\pi }}\vartheta^{-1/2}\text{exp}\left[-\frac{d\vartheta }{2}\right];\;\vartheta >0;k>0, \end{array}$$
(c) Gamma Prior
  (27) $$\begin{array}{c} h_{4}\left(\vartheta \right)=\frac{d^{k}}{\Gamma \left(k\right)}\vartheta^{k-1}\text{exp}\left[-d\vartheta \right];\;\vartheta >0;k,d>0. \end{array}$$

### 3.2. Posterior Density Functions

The posterior distribution for the shape parameter *ϑ* can be expressed as follows:

(28) $$\begin{array}{c} \pi \left(\vartheta |\underline{x}\right)=\frac{L\left(\vartheta ,\delta |\underline{x}\right)h\left(\vartheta \right)}{\int_{0}^{\infty }L\left(\vartheta ,\delta |\underline{x}\right)h\left(\vartheta \right)\text{d}\vartheta }. \end{array}$$

Combining the $L\left(|\underline{x}|\vartheta \right)$ in (4) and the prior distribution of extended Jeffrey's prior (16), chi-square prior (17), inverted Levy prior (18), and gamma prior (19). The posterior density of *ϑ* It can be found on respectively as follows:

(29) $$\begin{array}{c} \pi_{1}\;\left(\vartheta |\underline{x}\right)=\frac{T^{n-2c+1}}{\Gamma \left(n-2c+1\right)}\;\vartheta^{n-2c}\exp\left[-T\vartheta \right];\vartheta >0;c>0,\\ \pi_{2}\left(\vartheta |\underline{x}\right)=\frac{{\left(T+d/2\right)}^{n+k/2}}{\Gamma \left(n+k/2\right)}\vartheta^{n+k/2-1}\exp\left[-\left(T+\frac{d}{2}\right)\vartheta \right];\vartheta >0;k,d>0,\\ \pi_{3}\;\left(\vartheta |\underline{x}\right)=\frac{{\left(T+d/2\right)}^{n+1/2}}{\Gamma \left(n+1/2\right)}\;\vartheta^{n-1/2}\exp\left[-\left(T+\frac{d}{2}\right)\vartheta \right];\vartheta >0;k,d>0,\\ \pi_{4}\;\left(\vartheta |\underline{x}\right)=\frac{{\left(T+d\right)}^{n+k}}{\Gamma \left(n+k\right)}\;\vartheta^{n+k-1}\exp\left[-\left(T+d\right)\vartheta \right];\vartheta >0;k,d>0. \end{array}$$

## 4. Loss Functions

In Bayes estimation, we will consider three types of loss functions including SELL, LLF, and CLLF.

### 4.1. Squared Error Loss Function

The SELF is defined as follows [21]:

(30) $$\begin{array}{c} L\left(\hat{\vartheta },\vartheta \right)={\left(\hat{\vartheta }-\vartheta \right)}^{2}. \end{array}$$

The Bayes estimator of *ϑ* relative to SELF, signified by ${\hat{\vartheta }}_{\text{BSE}}$ is

(31) $$\begin{array}{c} {\hat{\vartheta }}_{\text{BSE}}=E_{h}\left(\vartheta |\underline{x}\right). \end{array}$$

### 4.2. LINEX Loss Function

The LINEX loss function for *ϑ* can be written as follows [22, 23]:

(32) $$\begin{array}{c} L\left(\hat{\vartheta },\vartheta \right)\propto \left[\exp\left[a\left(\hat{\vartheta }-\vartheta \right)\right]-a\left(\hat{\vartheta }-\vartheta \right)-1\right];a\neq 0. \end{array}$$

The Bayes estimator of *ϑ* relative to LLF, denoted by ${\hat{\vartheta }}_{BL}$ is

(33) $$\begin{array}{c} {\hat{\vartheta }}_{BL}=-\frac{1}{a}Ln\left[E_{\vartheta }\exp\left[-a\vartheta \right]\right];a\neq 0. \end{array}$$

Provided that *E*_*ϑ*_=exp[−*aϑ*] exists and is finite, where *E*_*ϑ*_ denotes the expected value.

### 4.3. Composite LINEX Loss Function

CLLF is given by the following formula [24].

(34) $$\begin{array}{c} L\left(\hat{\vartheta },\vartheta \right)=L_{a}\;\left(\hat{\vartheta },\vartheta \right)+L_{-a}\left(\hat{\vartheta },\vartheta \right)=\exp\left[-a\left(\hat{\vartheta },\vartheta \right)\right]+\exp\left[a\left(\hat{\vartheta }\;,\vartheta \right)\right]-2a>0. \end{array}$$

The Bayes estimator of *ϑ* relative to CLLF, denoted by ${\hat{\beta }}_{\text{BCL}}$, is

(35) $$\begin{array}{c} {\hat{\beta }}_{BCL}=\frac{1}{2a}\ln\left(\frac{E_{\vartheta }\left(\exp\left[a\vartheta \right]|\underline{x}\right)}{E_{\vartheta }\left(\exp\left[-a\vartheta \right]|\underline{x}\right)}\right). \end{array}$$

Provided that $E_{\vartheta }=\left(\text{exp}\left[a\vartheta \right]|\underline{x}\right)$ and $E_{\vartheta }\left(|\underline{x}\right)\left(\text{exp}\left[-a\vartheta \right]\right)$ exist and are finite.

## 5. Bayes Estimator

In this part, we estimate *ϑ*, using three various loss functions, including SELF, LLF, and CLLF. We assume four different prior distributions for *ϑ* including; extended Jeffrey's prior, chi-square prior, inverted Levy prior, and gamma prior [25–28].

### 5.1. Bayesian Estimator of *ϑ* under SELF

The Bayes estimates of *ϑ* relative to SELF depended on $\pi_{1}\left(\vartheta |\underline{x}\right)$ which is signified as ${\hat{\vartheta }}_{\text{BSE}1}$ and can be acquired by using equations (21) and (26) to be

(36) $$\begin{array}{c} {\hat{\vartheta }}_{BSE1}=E\left(\vartheta |\underline{x}\right)=\int_{0}^{\infty }\vartheta \pi_{1}\left(\vartheta |\underline{x}\right)d\vartheta ,\\ {\hat{\vartheta }}_{BSE1}=\int_{0}^{\infty }\frac{T^{n-2c+1}}{\Gamma \left(n-2c+1\right)}\vartheta^{n-2c+1}\exp\left[-T\vartheta \right]d\vartheta =\frac{n-2c+1}{T}. \end{array}$$

Likewise, we can obtain the Bayesian estimates of *ϑ* relative to SELF depending on $\pi_{2}\left(\vartheta |\underline{x}\right)$, $\pi_{3}\left(\vartheta |\underline{x}\right)$, and $\pi_{4}\left(\vartheta |\underline{x}\right)$, which are signified as ${\hat{\vartheta }}_{\text{BSE}2}$, ${\hat{\vartheta }}_{\text{BSE}3}$, and ${\hat{\vartheta }}_{\text{BSE}4}$ by using equations ((22) and (26)), ((23) and (26)) and ((24) and (26)), respectively, to be

(37) $$\begin{array}{c} {\hat{\vartheta }}_{BSE2}=E\left(\vartheta |\underline{x}\right)=\int_{0}^{\infty }\vartheta \pi_{2}\left(\vartheta |\underline{x}\right)\text{d}\vartheta ,\\ {\hat{\vartheta }}_{BSE2}=\int_{0}^{\infty }\frac{{\left(T+d/2\right)}^{n+k/2}}{\Gamma \left(n+k/2\right)}\vartheta^{n+k/2}\exp\left[-\left(T+\frac{d}{2}\right)\vartheta \right]\text{d}\vartheta =\frac{n+k/2}{T+d/2},\\ {\hat{\vartheta }}_{BSE3}=E\left(\vartheta |\underline{x}\right)=\int_{0}^{\infty }\vartheta \pi_{3}\left(\vartheta |\underline{x}\right)\text{d}\vartheta ,\\ {\hat{\vartheta }}_{BSE3}=\int_{0}^{\infty }\frac{{\left(T+d/2\right)}^{n+1/2}}{\Gamma \left(n+1/2\right)}\vartheta^{n+1/2}\exp\left[-\left(T+\frac{d}{2}\right)\vartheta \right]\text{d}\vartheta \;=\frac{\Gamma \left(n+3/2\right)}{\Gamma \left(n+1/2\right)\left(T+d/2\right)}, \end{array}$$

and

(38) $$\begin{array}{c} {\hat{\vartheta }}_{BSE4}=E\left(\vartheta |\underline{x}\right)=\int_{0}^{\infty }\vartheta \pi_{3}\left(\vartheta |\underline{x}\right)\text{d}\vartheta ,\\ {\hat{\vartheta }}_{BSE4}=E\left(\vartheta |\underline{x}\right)=\int_{0}^{\infty }\vartheta \pi_{3}\left(\vartheta |\underline{x}\right)\text{d}\vartheta ,\\ {\hat{\vartheta }}_{BSE4}=\int_{0}^{\infty }\frac{{\left(T+d\right)}^{n+k}}{\Gamma \left(n+k\right)}\vartheta^{n+k}\exp\left[-\left(T+d\right)\vartheta \right]\text{d}\vartheta \\ =\frac{n+k}{T+d}. \end{array}$$

### 5.2. Bayesian Estimator of *ϑ* under LLF

We can obtain the Bayes estimator of *ϑ* under the LLF depending on $\pi_{1}\left(\vartheta |\underline{x}\right)$ signified as ${\hat{\vartheta }}_{BL1}$ by using equations (21) and (28) as follows:

(39) $$\begin{array}{c} {\hat{\vartheta }}_{BL1}=-\frac{1}{a}Ln\left[E_{\vartheta }\exp\left[-a\vartheta \right]\right]=\int_{0}^{\infty }\exp\left[-a\vartheta \right]\pi_{1}\left(\vartheta |\underline{x}\right)\text{d}\vartheta ,\\ {\hat{\vartheta }}_{BL1}=-\frac{1}{a}\ln\int_{0}^{\infty }\exp\left[-a\vartheta \right]\frac{T^{n-2c+1}}{\Gamma \left(n-2c+1\right)}\vartheta^{n-2c}\exp\left[-T\vartheta \right]\text{d}\vartheta \\ =\frac{n-2c+1}{a}\ln\left(1+\frac{a}{T}\right). \end{array}$$

In the same way, the Bayes estimates of *ϑ* relative to LLF depended on $\pi_{2}\left(\vartheta |\underline{x}\right)$, $\pi_{3}\left(\vartheta |\underline{x}\right)$, and $\pi_{4}\left(\vartheta |\underline{x}\right)$, which are signified as ${\hat{\vartheta }}_{BL2}$, ${\hat{\vartheta }}_{BL3}$, and ${\hat{\vartheta }}_{BL4}$ by using equations ((22) and (28)) ((23) and (28)), and ((24) and (28)), respectively, to be

(40) $$\begin{array}{c} {\hat{\vartheta }}_{BL2}=-\frac{1}{a}Ln\left[E_{\vartheta }\exp\left[-a\vartheta \right]\right]=\int_{0}^{\infty }\exp\left[-a\vartheta \right]\pi_{2}\;\left(\vartheta |\underline{x}\right)d\vartheta ,\\ {\hat{\vartheta }}_{BL2}=-\frac{1}{a}\ln\int_{0}^{\infty }\exp\left[-a\vartheta \right]\frac{{\left(T+d/2\right)}^{n+k/2}}{\Gamma \left(n+k/2\right)}\;\vartheta^{n+k/2-1}\exp\left[-\left(T+\frac{d}{2}\right)\vartheta \right]d\vartheta \\ =\frac{n+0.5k}{a}\ln\left(1+\frac{a}{T+d/2}\right),\\ {\hat{\vartheta }}_{BL3}=-\frac{1}{a}Ln\left[E_{\vartheta }\exp\left[-a\vartheta \right]\right]=\int_{0}^{\infty }\exp\left[-a\vartheta \right]\pi_{3}\left(\vartheta |\underline{x}\right)d\vartheta ,\\ {\hat{\vartheta }}_{BL3}=-\frac{1}{a}\ln\int_{0}^{\infty }\exp\left[-a\vartheta \right]\frac{{\left(T+d/2\right)}^{n+1/2}}{\Gamma \left(n+1/2\right)}\vartheta^{n-1/2}\exp\left[-\left(T+\frac{d}{2}\right)\vartheta \right]d\vartheta \\ =\frac{n+0.5}{a}\ln\left(1+\frac{a}{T+d/2}\right), \end{array}$$

and

(41) $$\begin{array}{c} {\hat{\vartheta }}_{BL4}=-\frac{1}{a}Ln\left[E_{\vartheta }\exp\left[-a\vartheta \right]\right]=\int_{0}^{\infty }\exp\left[-a\vartheta \right]\pi_{4}\left(\vartheta |\underline{x}\right)\text{d}\vartheta ,\\ {\hat{\vartheta }}_{BL4}=-\frac{1}{a}\ln\int_{0}^{\infty }\exp\left[-a\vartheta \right]\frac{{\left(T+d\right)}^{n+k}}{\Gamma \left(n+k\right)}\vartheta^{n+k-1}\exp\left[-\left(T+d\right)\vartheta \right]\text{d}\vartheta \\ =\frac{n+k}{a}\ln\left(1+\frac{a}{T+d}\right). \end{array}$$

### 5.3. Bayesian Estimation of *ϑ* under CLLF

The Bayes estimate of *ϑ* under CLLF depended on $\pi_{1}\left(\vartheta |\underline{x}\right)$, which is signified as ${\hat{\vartheta }}_{\text{BCL}1}$ by using equations (21) and (30) to be

(42) $$\begin{array}{c} {\hat{\vartheta }}_{BCL1}=\frac{1}{2a}\ln\left(\frac{E_{\vartheta }\left(\exp\left[a\vartheta \right]|\underline{x}\right)}{E_{\vartheta }\left(\exp\left[-a\vartheta \right]|\underline{x}\right)}\right)=\frac{I_{1}}{I_{2}}, \end{array}$$

where:

(43) $$\begin{array}{c} I_{1}=E_{\vartheta }\left(\exp\left[a\vartheta \right]|\underline{x}\right)=\int_{0}^{\infty }\exp\left[a\vartheta \right]\pi_{1}\;\left(\vartheta |\underline{x}\right)\text{d}\vartheta \\ =\int_{0}^{\infty }\exp\left[a\vartheta \right]\frac{T^{n-2c+1}}{\Gamma \left(n-2c+1\right)}\vartheta^{n-2c}\exp\left[-T\vartheta \right]\text{d}\vartheta \\ ={\left(\frac{T}{T-a}\right)}^{n-2c+1}, \end{array}$$

and

(44) $$\begin{array}{c} I_{2}=E_{\vartheta }\left(\exp\left[-a\vartheta \right]|\underline{x}\right)=\int_{0}^{\infty }\exp\left[-a\vartheta \right]\pi_{1}\left(\vartheta |\underline{x}\right)d\vartheta \\ =\int_{0}^{\infty }\exp\left[-a\vartheta \right]\frac{T^{n-2c+1}}{\Gamma \left(n-2c+1\right)}\vartheta^{n-2c}\exp\left[-T\vartheta \right]d\vartheta \\ ={\left(\frac{T}{T+a}\right)}^{n-2c+1}. \end{array}$$

Therefore, the Bayes estimation of parameter *ϑ* is

(45) $$\begin{array}{c} {\hat{\vartheta }}_{BCL1}=\frac{1}{2a}\ln\left[\frac{{\left(T/T-a\right)}^{n-2c+1}}{{\left(T/T+a\right)}^{n-2c+1}}\right]=\frac{n-2c+1}{2a}\ln\left(\frac{T+a}{T-a}\right). \end{array}$$

Similarly, the Bayesian estimates of *ϑ* under CLLF depended on $\pi_{2}\left(\vartheta |\underline{x}\right)$, $\pi_{3}\left(\vartheta |\underline{x}\right)$, and $\pi_{4}\left(\vartheta |\underline{x}\right)$, which are signified as ${\hat{\vartheta }}_{\text{BCL}2}$, ${\hat{\vartheta }}_{\text{BCL}3}$, and ${\hat{\vartheta }}_{\text{BCL}4}$ by using equations ((22) and (30)), ((23) and (30)), and ((24) and (30)), respectively, to be

(46) $$\begin{array}{c} {\hat{\vartheta }}_{BCL2}=\frac{1}{2a}\ln\left(\frac{E_{\vartheta }\left(\exp\left[a\vartheta \right]|\underline{x}\right)}{E_{\vartheta }\left(\exp\left[-a\vartheta \right]|\underline{x}\right)}\right)=\frac{I_{3}}{I_{4}}, \end{array}$$

where

(47) $$\begin{array}{c} I_{3}=E_{\vartheta }\left(\exp\left[a\vartheta \right]|\underline{x}\right)=\int_{0}^{\infty }\exp\left[a\vartheta \right]\pi_{2}\left(\vartheta |\underline{x}\right)d\vartheta \\ =\int_{0}^{\infty }\exp\left[a\vartheta \right]\frac{{\left(T+d/2\right)}^{n+k/2}}{\Gamma \left(n+k/2\right)}\;\vartheta^{n+k/2-1}\exp\left[-\left(T+\frac{d}{2}\right)\vartheta \right]d\vartheta \\ ={\left(\frac{T+d/2}{T+d/2-a}\right)}^{n+k/2}, \end{array}$$

and

(48) $$\begin{array}{c} I_{4}=E_{\vartheta }\left(\exp\left[-a\vartheta \right]|\underline{x}\right)=\int_{0}^{\infty }\exp\left[-a\vartheta \right]\pi_{2}\left(\vartheta |\underline{x}\right)d\vartheta \\ =\int_{0}^{\infty }\exp\left[-a\vartheta \right]\frac{{\left(T+d/2\right)}^{n+k/2}}{\Gamma \left(n+k/2\right)}\vartheta^{n+k/2-1}\exp\left[-\left(T+\frac{d}{2}\right)\vartheta \right]d\vartheta \\ ={\left(\frac{T+d/2}{T+d/2+a}\right)}^{n+k/2}, \end{array}$$

So

(49) $$\begin{array}{c} {\hat{\vartheta }}_{BCL2}=\frac{1}{2a}\ln\left[\frac{{\left(\left(T+d/2\right)/\left(T+d/2-a\right)\right)}^{n+k/2}\;}{{\left(\left(T+d/2\right)/\left(T+d/2+a\right)\right)}^{n+k/2}}\right]=\frac{n+k/2}{2a}\ln\left(\frac{T+\left(d/2\right)+a}{T+\left(d/2\right)-a}\right), \end{array}$$

and

(50) $$\begin{array}{c} {\hat{\vartheta }}_{BCL3}=\frac{1}{2a}\ln\left(\frac{E_{\vartheta }\left(\exp\left[a\vartheta \right]|\underline{x}\right)}{E_{\vartheta }\left(\exp\left[-a\vartheta \right]|\underline{x}\right)}\right)=\frac{I_{5}}{I_{6}}, \end{array}$$

where

(51) $$\begin{array}{c} I_{5}=E_{\vartheta }\left(\exp\left[a\vartheta \right]|\underline{x}\right)=\int_{0}^{\infty }\exp\left[a\vartheta \right]\pi_{3}\left(\vartheta |\underline{x}\right)d\vartheta \\ =\int_{0}^{\infty }\exp\left[a\vartheta \right]\frac{{\left(T+d/2\right)}^{n+1/2}}{\Gamma \left(n+1/2\right)}\vartheta^{n-1/2}\exp\left[-\left(T+\frac{d}{2}\right)\vartheta \right]d\vartheta \\ ={\left(\frac{T+d/2}{T+\left(d/2\right)-a}\right)}^{n+1/2}, \end{array}$$

and

(52) $$\begin{array}{c} I_{6}=E_{\vartheta }\left(\exp\left[-a\vartheta \right]|\underline{x}\right)=\int_{0}^{\infty }\exp\left[-a\vartheta \right]\pi_{3}\left(\vartheta |\underline{x}\right)d\vartheta \\ =\int_{0}^{\infty }\exp\left[-a\vartheta \right]\frac{{\left(T+d/2\right)}^{n+1/2}}{\Gamma \left(n+1/2\right)}\vartheta^{n-1/2}\exp\left[-\left(T+\frac{d}{2}\right)\vartheta \right]d\vartheta \\ ={\left(\frac{T+d/2}{T+d/2+a}\right)}^{n+1/2}, \end{array}$$

So

(53) $$\begin{array}{c} {\hat{\vartheta }}_{BCL3}=\frac{1}{2a}\ln\left[\frac{{\left(\left(T+d/2\right)/\left(T+d/2-a\right)\right)}^{n+1/2}\;}{{\left(\left(T+d/2\right)/\left(T+d/2+a\right)\right)}^{n+1/2}}\;\right]=\frac{n+1/2}{2a}\ln\left(\frac{T+d/2+a}{T+d/2-a}\right),\\ {\hat{\vartheta }}_{BCL4}=\frac{1}{2a}\ln\left(\frac{E_{\vartheta }\left(\exp\left[a\vartheta \right]|\underline{x}\right)}{E_{\vartheta }\left(\exp\left[-a\vartheta \right]|\underline{x}\right)}\right)=\frac{I_{7}}{I_{8}}, \end{array}$$

where

(54) $$\begin{array}{c} I_{7}=E_{\vartheta }\left(\exp\left[a\vartheta \right]|\underline{x}\right)=\int_{0}^{\infty }\exp\left[a\vartheta \right]\pi_{4}\left(\vartheta |\underline{x}\right)d\vartheta \\ =\int_{0}^{\infty }\exp\left[a\vartheta \right]\frac{{\left(T+d\right)}^{n+k}}{\Gamma \left(n+k\right)}\vartheta^{n+k-1}\exp\left[-\left(T+d\right)\vartheta \right]d\vartheta \\ ={\left(\frac{T+d}{T+d-a}\right)}^{n+k}, \end{array}$$

and

(55) $$\begin{array}{c} I_{8}=E_{\vartheta }\left(\exp\left[-a\vartheta \right]|\underline{x}\right)\\ =\int_{0}^{\infty }\exp\left[-a\vartheta \right]\pi_{4}\left(\vartheta |\underline{x}\right)d\vartheta \\ =\int_{0}^{\infty }\exp\left[-a\vartheta \right]\frac{{\left(T+d\right)}^{n+k}}{\Gamma \left(n+k\right)}\vartheta^{n+k-1}\exp\left[-\left(T+d\right)\vartheta \right]d\vartheta \\ ={\left(\frac{T+d}{T+d+a}\right)}^{n+k}. \end{array}$$

Therefore, the Bayes estimation of parameter *ϑ* is

(56) $$\begin{array}{c} {\hat{\vartheta }}_{BCL4}=\frac{1}{2a}\ln\left[\frac{{\left(T+d/T+d/2-a\right)}^{n+k}\;}{{\left(T+d/T+d+a\right)}^{n+k}}\right]=\frac{n+k}{2a}\ln\left(\frac{T+d+a}{T+d-a}\right). \end{array}$$

## 6. Simulation Study and Results

In this part, a simulation study has been conducted to assess and examine the behavior of Classical methods and Bayes estimators for the shape parameter of LD under different cases. The following steps of the simulation are as follows:

(1) Set the true values for the parameters of LD which are varied into three cases to observe their effect on the estimates when *δ* > *ϑ*, *δ*=*ϑ*, and *δ* < *ϑ* “case I (*δ*=2, *ϑ*=1.5 ), case II (*δ*=2, *ϑ*=2 ) and case III (*δ*=2, *ϑ*=2.5 )”
(2) Determine the sample size *n*=20,40,60,80 and 100
(3) Determine the value *λ* = 0.75, *c*=0.5, (*K*, *d*)=(0.6, 0.2) and *a*=0.5, 1 and 1.5
(4) For a given sample size *n*, generate *x*_1_, *x*_2_,…*x*_*n*_ by using the following formula: *x*_*i*_=*δ*[(1 − *U*_*i*_)^−1/*ϑ*^ − 1], *i* = 1,   …,  *n*, where *U*_*i*_ is uniform (0,1)
(5) Classical methods estimation, ${\hat{\vartheta }}_{MLE}$, ${\hat{\vartheta }}_{\text{OLS}}$ , ${\hat{\vartheta }}_{\text{Rid}}$, ${\hat{\vartheta }}_{\text{UMVUE}}$, and ${\hat{\vartheta }}_{\text{Med}}$ of *ϑ* are computed from equations (6), (10), (13)–(15), respectively
(6) Under SELL and based on *h*_1_(*ϑ*), *h*_2_(*ϑ*)*h*_3_(*ϑ*) and *h*_4_(*ϑ*) priors, Bayesian estimation, ${\hat{\vartheta }}_{\text{BSE}1}$, ${\hat{\vartheta }}_{\text{BSE}2}$, ${\hat{\vartheta }}_{\text{BSE}3}$ and ${\hat{\vartheta }}_{\text{BSE.\$}}$, of *ϑ* are computed from equations (31), (32), (33), and (34), respectively
(7) Under LLF and based on *h*_1_(*ϑ*), *h*_2_(*ϑ*), *h*_3_(*ϑ*) and *h*_4_(*ϑ*) priors, Bayesian estimation, ${\hat{\vartheta }}_{BL1}$, ${\hat{\vartheta }}_{BL2}$, ${\hat{\vartheta }}_{BL3}$ and ${\hat{\vartheta }}_{BL4}$, of *ϑ* are computed from equations (35), (36), (37), and (38), respectively
(8) Under CLLF and based on *h*_1_(*ϑ*), *h*_2_(*ϑ*)*h*_3_(*ϑ*) and *h*_4_(*ϑ*) priors, ${\hat{\vartheta }}_{BCL1}$, ${\hat{\vartheta }}_{BCL2}$, ${\hat{\vartheta }}_{\text{BCL}3}$ and ${\hat{\vartheta }}_{\text{BCL}4}$, of *ϑ* are calculated from equations (40), (42), (44), and (46), respectively
(9) Steps 4 to 8 are replicated 10,000 times. The (MSE's) for all Estimates of the parameter  *ϑ* are obtained, where
  (57) $$\begin{array}{c} \text{MSE }\left(\hat{\vartheta }\right)=\frac{1}{10000}\sum_{i=1}^{10000}{\left({\hat{\vartheta }}_{i}-\vartheta \right)}^{2}. \end{array}$$

The results are displayed in the following Tables 1–5.

## 7. Conclusions and Recommendations

In this paper, the Ridge Regression method was employed to estimate the shape parameter of LD. Besides, researchers made a Monte Carlo simulation to test the performance of the Ridge Regression method. Then, compared the Ridge Regression estimator with the other estimators, including MLE, OLS, UMVUE, M.M, and Bayesian estimators based on SELF, LLF, and CLLF. However, the major observations are identified in the following points:

1. Among classical estimators, in Table 1, the performance of the UMVUE was shown as better than other estimators: “MLE, OLS, Ridge, and M.M estimators” in all different cases and all samples sizes. Whereas the performance of Ridge Regression was better than MLE. Estimator especially for a small sample size (*n* = 10). In the meanwhile, the results showed that the performance of the Ridge estimator was better than OLS estimator in all different cases and sample sizes.
2. With Bayes estimators, gamma prior records full appearance as best prior based on LLF and CLLF for all different cases and all sample sizes. As well as that is true under SELF with *δ* = *ϑ* and *δ* < *ϑ* , while extended Jeffrey's prior record as best prior based on SELF when *δ* > *ϑ*.
3. The MSE values associated with each of the classical and Bayes estimate “corresponding to each prior and every loss function” reduces with the increase in the sample size. Also, the results show a convergence between most of the estimators to increase the sample sizes and this conforms to the statistical theory.
4. For all cases and all sample sizes, LLF (*a* = 1.5) records full appearance as the best loss function associated with Bayes estimates corresponding to gamma prior.
5. According to the results, MSE values of all classical and Bayes estimators of shape parameters are decreasing as the shape parameter value increase.

## Figures and Tables

**Table 1:** MSE values for non-bayes estimators of *ϑ*.

Cases	*n*	${\hat{\vartheta }}_{\text{MLE}}$	${\hat{\vartheta }}_{\text{OLS}}$	${\hat{\vartheta }}_{\text{Rid}}$	${\hat{\vartheta }}_{\text{UMVUE}}$	${\hat{\vartheta }}_{\text{Med}}$
*I*	20	0.1457	0.2021	0.1350	0.1249	0.3042
	40	0.0645	0.1005	0.0870	0.0599	0.1440
	60	0.0408	0.0689	0.0631	0.0388	0.0873
	80	0.0302	0.0531	0.0501	0.0291	0.0665
	100	0.0237	0.0417	0.0399	0.0229	0.0507

II	20	0.2551	0.3585	0.2434	0.2196	0.5406
	40	0.1126	0.1814	0.1521	0.1047	0.2412
	60	0.0702	0.1224	0.1105	0.0667	0.1528
	80	0.0526	0.0942	0.0882	0.0507	0.1148
	100	0.0412	0.0770	0.0732	0.0399	0.0878

III	20	0.3995	0.5743	0.4357	0.3462	0.7967
	40	0.1743	0.2818	0.2455	0.1621	0.3770
	60	0.1116	0.1864	0.1735	0.1062	0.2475
	80	0.0807	0.1478	0.1416	0.0778	0.1775
	100	0.0668	0.1215	0.1175	0.0649	0.1440

**Table 2:** MSE values for bayes estimators of *ϑ* with extended Jeffrey's prior.

Cases	*n*	${\hat{\vartheta }}_{\text{BSE}1}$	${\hat{\vartheta }}_{BL1}$			${\hat{\vartheta }}_{\text{BCL}1}$		
			*a*=0.5	*a*=1	*a*=1.5	*a*=0.5	*a*=1	*a*=1.5
*I*	20	0.1457	0.1298	0.1177	0.1081	0.1464	0.1476	0.1511
	40	0.0645	0.0611	0.0560	0.0532	0.0645	0.0621	0.0614
	60	0.0408	0.0393	0.0371	0.0371	0.0408	0.0397	0.0408
	80	0.0302	0.0294	0.0292	0.0275	0.0303	0.0307	0.0296
	100	0.0237	0.0232	0.0230	0.0223	0.0237	0.0239	0.0237

II	20	0.2551	0.2200	0.1961	0.1803	0.2574	0.2628	0.2776
	40	0.1126	0.1050	0.0967	0.0952	0.1128	0.1104	0.1151
	60	0.0702	0.0670	0.0655	0.0632	0.0703	0.0716	0.0708
	80	0.0526	0.0508	0.0504	0.0480	0.0526	0.0536	0.0526
	100	0.0412	0.0400	0.0397	0.0388	0.0412	0.0419	0.0415

III	20	0.3995	0.3347	0.2922	0.2727	0.4051	0.4189	0.4535
	40	0.1743	0.1601	0.1517	0.1441	0.1748	0.1780	0.1794
	60	0.1116	0.1054	0.1012	0.0985	0.1117	0.1126	0.1121
	80	0.0807	0.0774	0.0729	0.0723	0.0808	0.0786	0.0805
	100	0.0668	0.0646	0.0600	0.0596	0.0668	0.0636	0.0646

**Table 3:** MSE values for bayes estimators of *ϑ* with chi-square prior.

Cases	*n*	${\hat{\vartheta }}_{BSE1}$	${\hat{\vartheta }}_{BL1}$			${\hat{\vartheta }}_{BCL1}$		
			*a*=0.5	*a*=1	*a*=1.5	*a*=0.5	*a*=1	*a*=1.5
*I*	20	0.1468	0.1305	0.1177	0.1077	0.1476	0.1486	0.1522
	40	0.0648	0.0613	0.0561	0.0531	0.0649	0.0625	0.0617
	60	0.0410	0.0395	0.0371	0.0370	0.0410	0.0399	0.0410
	80	0.0304	0.0295	0.0292	0.0275	0.0304	0.0309	0.0297
	100	0.0238	0.0232	0.0231	0.0223	0.0238	0.0240	0.0238

II	20	0.2529	0.2177	0.1933	0.1771	0.2552	0.2602	0.2746
	40	0.1124	0.1045	0.0961	0.0944	0.1126	0.1101	0.1149
	60	0.0702	0.0668	0.0653	0.0629	0.0702	0.0715	0.0708
	80	0.0526	0.0507	0.0502	0.0479	0.0526	0.0536	0.0526
	100	0.0412	0.0400	0.0397	0.0386	0.0412	0.0419	0.0415

III	20	0.3895	0.3265	0.2849	0.2658	0.3948	0.4077	0.4405
	40	0.1726	0.1584	0.1499	0.1423	0.1731	0.1762	0.1776
	60	0.1109	0.1047	0.1005	0.0977	0.1110	0.1119	0.1114
	80	0.0804	0.0770	0.0725	0.0719	0.0804	0.0783	0.0801
	100	0.0666	0.0643	0.0598	0.0593	0.0666	0.0634	0.0643

**Table 4:** MSE values for bayes estimators of *ϑ* with inverted Levy prior.

Cases	*n*	${\hat{\vartheta }}_{BSE1}$	${\hat{\vartheta }}_{BL1}$			${\hat{\vartheta }}_{BCL1}$		
			*a*=0.5	*a*=1	*a*=1.5	*a*=0.5	*a*=1	*a*=1.5
*I*	20	0.1528	0.1352	0.1210	0.1100	0.1536	0.1544	0.1583
	40	0.0662	0.0624	0.0569	0.0536	0.0663	0.0638	0.0630
	60	0.0416	0.0400	0.0375	0.0373	0.0416	0.0405	0.0416
	80	0.0307	0.0298	0.0294	0.0277	0.0307	0.0312	0.0300
	100	0.0240	0.0234	0.0232	0.0224	0.0240	0.0242	0.0240

II	20	0.2630	0.2248	0.1974	0.1793	0.2654	0.2703	0.2858
	40	0.1146	0.1062	0.0972	0.0951	0.1149	0.1124	0.1174
	60	0.0712	0.0676	0.0658	0.0631	0.0712	0.0726	0.0717
	80	0.0531	0.0512	0.0505	0.0481	0.0532	0.0541	0.0532
	100	0.0416	0.0403	0.0399	0.0387	0.0416	0.0422	0.0418

III	20	0.4043	0.3358	0.2892	0.2662	0.4099	0.4234	0.4581
	40	0.1760	0.1607	0.1511	0.1425	0.1765	0.1798	0.1813
	60	0.1124	0.1057	0.1011	0.0977	0.1126	0.1135	0.1128
	80	0.0812	0.0776	0.0728	0.0720	0.0813	0.0791	0.0811
	100	0.0671	0.0647	0.0599	0.0594	0.0671	0.0639	0.0649

**Table 5:** MSE values for bayes estimators of *ϑ* with gamma prior.

Cases	*n*	${\hat{\vartheta }}_{\text{BSE}1}$	${\hat{\vartheta }}_{BL1}$			${\hat{\vartheta }}_{\text{BCL}1}$		
			*a*=0.5	*a*=1	*a*=1.5	*a*=0.5	*a*=1	*a*=1.5
*I*	20	0.1482	0.1314	0.1179	0.1075	0.1489	0.1498	0.1534
	40	0.0652	0.0615	0.0562	0.0530	0.0653	0.0629	0.0621
	60	0.0412	0.0396	0.0372	0.0370	0.0412	0.0401	0.0412
	80	0.0305	0.0296	0.0293	0.0275	0.0305	0.0310	0.0298
	100	0.0239	0.0233	0.0231	0.0223	0.0239	0.0240	0.0239

II	20	0.2510	0.2156	0.1907	0.1742	0.2531	0.2578	0.2720
	40	0.1121	0.1041	0.0956	0.0937	0.1123	0.1099	0.1147
	60	0.0701	0.0667	0.0651	0.0626	0.0702	0.0715	0.0707
	80	0.0526	0.0507	0.0501	0.0477	0.0526	0.0535	0.0526
	100	0.0412	0.0400	0.0396	0.0385	0.0412	0.0419	0.0415

III	20	0.3801	0.3187	0.2779	0.2591	0.3851	0.3971	0.4284
	40	0.1709	0.1567	0.1482	0.1405	0.1714	0.1745	0.1759
	60	0.1102	0.1040	0.0997	0.0969	0.1104	0.1113	0.1107
	80	0.0800	0.0767	0.0721	0.0714	0.0801	0.0780	0.0798
	100	0.0663	0.0641	0.0595	0.0591	0.0664	0.0632	0.0641

## Data Availability

The data used to support the findings of this study are available from the corresponding author upon request.
